# Integrative Single-Cell Transcriptomics and Network Modeling Reveal Modular Regulators of Sheep Zygotic Genome Activation

**DOI:** 10.3390/biology14060676

**Published:** 2025-06-11

**Authors:** Xiaopeng Li, Peng Niu, Kai Hu, Xueyan Wang, Fei Huang, Pengyan Song, Qinghua Gao, Di Fang

**Affiliations:** 1 College of Animal Science and Technology, Tarim University, Alar 843300, China; miexiaochi@163.com (X.L.);; 2 Key Laboratory of Livestock and Forage Resources Utilization Around Tarim, Ministry of Agriculture and Rural Affairs, Alar 843300, China; 3 College of Life Science and Technology, Tarim University, Alar 843300, China

**Keywords:** zygotic genome activation, sheep embryos, single cell transcriptomics, WGCNA, Louvain algorithm

## Abstract

Zygotic genome activation (ZGA) marks the critical transition from reliance on maternal transcripts to the initiation of embryonic transcription early in development. Despite extensive characterization in model species, the regulatory framework of ZGA in sheep remains poorly defined. Here, we applied single-cell RNA sequencing (Smart-seq2) to in vivo- and in vitro-derived sheep embryos at the 8-, 16-, and 32-cell stages. Differential expression analysis revealed 114, 1628, and 1465 genes altered in the 8- vs. 16-, 16- vs. 32-, and 8- vs. 32-cell transitions, respectively, with the core pluripotency factors *SOX2*, *NANOG*, *POU5F1*, and *KLF4* upregulated during major ZGA. To uncover coordinated regulatory modules, we constructed a weighted gene co-expression network using WGCNA, identifying the MEred module as most tightly correlated with developmental progression (r = 0.48, *p* = 8.6 × 10^−14^). The integration of MERed genes into the STRING v11 protein–protein interaction network furnished a high-confidence scaffold for community detection. Louvain partitioning delineated two discrete communities: Community 0 was enriched in ER–Golgi vesicle-mediated transport, transmembrane transport, and cytoskeletal dynamics, suggesting roles in membrane protein processing, secretion, and early signaling; Community 1 was enriched in G2/M cell-cycle transition and RNA splicing/processing, indicating a coordinated network for accurate post-ZGA cell division and transcript maturation. Together, these integrated analyses reveal a modular regulatory architecture underlying sheep ZGA and provide a framework for dissecting early embryonic development in this species.

## Simple Summary

Zygotic genome activation (ZGA) marks the shift from maternal transcripts to embryonic transcription and is essential for early development. Here, we applied Smart-seq2 single-cell RNA-seq to 8-, 16-, and 32-cell sheep embryos (in vivo and in vitro) and then combined differential expression (|log_2_FC| > 1, *p*.adj < 0.05), WGCNA, and Louvain community detection to reveal two regulatory modules: one centered on ER–Golgi vesicle transport/cytoskeletal dynamics, and another on G2/M transition/RNA splicing. Our findings delineate a modular framework of sheep ZGA and suggest targets for improving reproductive efficiency.

## 1. Introduction

Zygotic genome activation (ZGA) is a crucial transcriptional event in mammalian development, signifying the transition from maternal mRNA and proteins to transcription driven by the embryonic genome [1]. During the early stages of embryonic development, transcription remains largely inactive, and the embryo primarily relies on maternal transcripts and proteins [2,3,4]. ZGA occurs in two distinct phases: minor ZGA and major ZGA [5,6]. Minor ZGA, characterized by low-level gene expression, establishes the groundwork for major ZGA [7]. During minor ZGA, RNA polymerase II (RNA Pol II) accumulates at gene promoters with high GC contents. In contrast, major ZGA is marked by a significant increase in transcriptional activity [8,9]. The timing of ZGA varies among species, occurring at the 1-cell stage in mice, the late 2-cell stage in cattle and humans, and the 8- vs 16-cell stage in sheep [10,11,12]. Epigenetic modifications, notably histone H3 lysine 4 trimethylation (H3K4me3), have emerged as critical regulators of ZGA. Broad H3K4me3 domains established in oocytes are refined into promoter-centric peaks during major ZGA, thereby facilitating transcriptional competence [13]. In that same study, Kdm5b knockdown was shown to enhance H3K4me3 deposition at LINE1 and ERVL elements, which correlated with increased RNA polymerase II occupancy and TE expression during ZGA [13]. These findings underscore a dual role for H3K4me3 in licensing both gene- and TE-driven transcription during early development [13].

Despite these advances, the extent to which epigenetic and transcriptional regulators are organized into coordinated networks during sheep ZGA remains poorly understood. In particular, it is unknown whether sheep embryos deploy discrete co-expression modules to orchestrate minor and major ZGA or how H3K4me3–Pol II interactions at TEs integrate into these networks. To address these gaps, we performed Smart-seq2 single cell RNA sequencing on in vivo- and in vitro-derived sheep embryos at the 8-, 16-, and 32-cell stages. By combining differential expression and weighted gene co-expression network analyses, we aim to (i) delineate the modular transcriptional architecture of sheep ZGA, (ii) identify key signaling pathways and hub regulators, and (iii) explore the interplay between epigenetic reprogramming and TE activation.

This work establishes a comprehensive framework for understanding the molecular drivers of early embryonic development in sheep.

## 2. Materials and Methods

In this experiment, all embryo samples were collected from the Key Laboratory of Livestock and Grassland Resources Utilization in the Tarim Basin (Tarim University Animal Experiment Station, Xinjiang, China). The ovaries were sourced from Xiyu Livestock Co., Ltd., located in Aksu, Xinjiang, China, during routine slaughter. A total of 12 single-embryo samples were used for Smart-seq2-based transcriptome sequencing, including embryos at the 8-cell, 16-cell, and 32-cell stages. For each developmental stage, four embryos were analyzed: two in vivo-derived and two in vitro-derived. All embryos were morphologically normal and evaluated based on cell number, symmetry, and zona pellucida integrity under a stereomicroscope.

### 2.1. Chemicals and Culture Media

All chemicals were purchased from Sigma-Aldrich (Oakville, ON, Canada), unless stated otherwise.

### 2.2. Animals and Sample Collection

#### 2.2.1. In Vivo Embryo Production

Healthy female sheep, aged 2 to 4 years, were selected as donors to ensure high oocyte quality. Superovulation was initiated during the luteal phase using a decreasing dose of follicle-stimulating hormone (FSH) via intramuscular injection over 3–4 days. Concurrently with the final FSH injection, 2 mg of prostaglandin F_2_α (PGF_2_α) was administered intramuscularly to induce luteolysis and estrus. Estrous behavior was closely monitored 36–48 h after PGF_2_α injection, and artificial insemination (AI) was performed 12 h post-estrus detection. Embryos were recovered non-surgically on day 6 post-insemination, with mild sedation applied to minimize stress. The uterus was flushed with sterile saline, and the recovered fluid was transferred to a Petri dish. Under a stereomicroscope, embryos at the 8-cell and 16-cell stages were selected and evaluated based on morphological characteristics, including cell number, symmetry, and zona pellucida integrity.

#### 2.2.2. In Vitro Embryo Production

**Oocyte Collection and In Vitro Maturation (IVM)**

Ovaries were obtained from abattoir-sourced sheep and transported in sterile saline at 38 °C containing 100 IU/mL penicillin and 100 µg/mL streptomycin. Upon arrival, ovaries were rinsed in saline and 70% ethanol and then maintained at 37 °C.

Follicles (2–6 mm diameter) were aspirated using a sterile 5 mL syringe; cumulus–oocyte complexes (COCs) were selected under a stereomicroscope. Grade A COCs exhibited ≥3 compact cumulus cell layers; Grade B COCs had 2–3 layers with a uniform cytoplasm.

Selected COCs were rinsed in pre-equilibrated maturation medium and cultured in 80 µL droplets (30 COCs/droplet) under mineral oil at 38.5 °C, 5% CO_2_, and saturated humidity for 24 h.

After maturation, cumulus cells were removed by incubation in PBS containing 0.1% hyaluronidase; oocyte maturity was confirmed based on first polar body extrusion.

**In Vitro Fertilization (IVF)**

Ram semen was collected via electroejaculation, diluted 1:4 in diluent, and incubated at 37 °C. Capacitation was performed according to the manufacturer’s protocol. After 30 min at 38.5 °C, sperm were washed twice at 800 × *g* and resuspended to 1 × 10^6^ sperm/mL. Mature oocytes were washed in fertilization medium and co-incubated with sperm (≤15 oocytes/50 µL droplet) at 38.5 °C, 5% CO_2_, and 100% humidity for 18 h. At 24 h post-insemination, cumulus cells were removed by gentle pipetting in PBS, and presumptive zygotes were washed twice in equilibrated SOF before culture.

**In Vitro Culture (IVC)**

Presumptive zygotes were cultured in synthetic oviduct fluid (SOF) supplemented with 0.5× essential and non-essential amino acids, 3 mg/mL bovine serum albumin, and 5% fetal bovine serum. Seventy-microliter SOF droplets under mineral oil were equilibrated at 38.5 °C, 5% CO_2_, and saturated humidity for ≥4 h. No more than 15 embryos were cultured per droplet. Cleavage was assessed at 48 h (2–4-cell rate) and 72 h (8–16-cell rate) post-insemination. Half of the medium was replaced every 48 h (days 2, 4, and 6) without disturbing the embryos.

Morula formation was evaluated on day 5 (120 h), based on compaction and the cell number (16–32 cells). Blastocyst development was recorded on day 7 (168 h); blastocysts were graded (1–3) based on blastocoel expansion and inner cell mass quality according to International Embryo Technology Society standards. All procedures were conducted under sterile conditions and recorded with time-stamped observations for subsequent analysis.

### 2.3. RNA Extraction and High-Throughput Sequencing

Two embryos were collected from each of the in vivo and in vitro groups at the 8-cell, 16-cell, and 32-cell stages, resulting in four embryos per developmental stage (total = 12 samples). Each embryo was immediately placed in 10 µL of lysis buffer containing RNase inhibitors (RNeasy Micro Kit, Qiagen, Hilden, Germany, Cat. No. 74004) to preserve RNA integrity. Total RNA was extracted according to the manufacturer’s protocol. The RNA concentration and purity were measured on a NanoDrop 2000 spectrophotometer (Thermo Fisher Scientific, San Diego, CA, USA), and the RNA integrity was assessed on an Agilent 2100 Bioanalyzer (Agilent Technologies, Santa Clara, CA, USA); all samples exhibited an RIN ≥ 8.0.

First-strand cDNA synthesis was performed using the PrimeScript RT Reagent Kit (Takara, Shiga, Japan, Cat. No. RR037A) following the manufacturer’s protocol. RNA libraries were prepared using the NEBNext Ultra II RNA Library Prep Kit for Illumina (New England Biolabs, Ipswich, MA, USA, Cat. No. E7770) with poly(A) enrichment. Sequencing was conducted on an Illumina NovaSeq 6000 platform (Illumina, San Diego, CA, USA) with paired-end 150 bp reads.

### 2.4. RNA Data Analysis

The raw sequencing reads were first evaluated with FastQC v0.12.1 (https://www.bioinformatics.babraham.ac.uk/projects/fastqc/, accessed on 5 June 2025) to assess the per-base quality, GC content, and adapter contamination. Adapter and low-quality sequences were then removed using Trimmomatic v0.39 [14] with the following parameters: (1) Phred33 quality encoding; (2) trimming of bases with quality <3 from both the 5′ and 3′ ends; (3) application of a 5-bp sliding window to cut when the average quality fell below 20; (4) removal of the first 13 bp to eliminate Smart-seq2 template-switching bias; and (5) discarding of any reads shorter than 36 bp. The resulting clean reads were aligned to the sheep reference genome (Oar_v4.0) using HISAT2 v2.2.1 [15], sorted with SAMtools, and quantified with FeatureCounts v2.0.6 [16] to produce a gene-by-sample count matrix. Differential expression analysis was carried out in R (v4.4.0) using DESeq2 v1.34.0 [17], applying the Benjamini–Hochberg correction; genes with an adjusted *p*-value (*p*.adj) < 0.05 and |log_2_ fold-change| > 1 were defined as differentially expressed and used for subsequent analyses.

### 2.5. Weighted Gene Co-Expression Network Analysis (WGCNA)

We constructed a gene co-expression network using the WGCNA R package in the R environment (version 4.4.0) [18]. First, we selected the top 5000 genes with the greatest variation in gene expression (measured based on the median absolute deviation, MAD), and these genes were used to build the co-expression network. To construct the weighted co-expression network, we evaluated different soft threshold values (β) and determined the optimal value. The pickSoftThreshold function was used to identify the best soft threshold, which amplifies strong correlations between genes and penalizes weak correlations [19]. Based on the calculated optimal soft threshold (β = 8), we proceeded with the network construction. To explore the correlation between different gene modules and various cell developmental stages, we utilized the module eigengene (ME) values and the sample phenotype data and visualized the correlations between the modules and phenotypes using a heatmap.

### 2.6. Louvain Community Detection

We constructed a gene co-expression network based on the PPI network obtained from the MERed module, which was derived from the WGCNA analysis. The purpose was to identify potential regulatory modules involved in zygotic genome activation (ZGA) in sheep. Experimental protein–protein interaction (PPI) data were initially processed to generate an undirected weighted graph G=(V,E), where each node represents a gene and the weight $A_{\mathrm{ij}}$ assigned to an edge indicates the interaction strength between gene i and gene j. The degree of node i is defined as

$$K_{i}=\sum_{j}A_{ij}$$

and the total weight of all edges is given by

$$m=\frac{1}{2}\sum_{i,j}A_{ij.}$$

Since the raw PPI network comprises a considerable number of low-degree nodes that often participate in only a few interactions and may be confounded by experimental noise, a filtering step was introduced during the initial network construction. Based on a statistical analysis of the node degree distribution—where the maximum node degree observed was approximately 213—we set a degree threshold of degree_threshold = 200, retaining only those nodes with $k_{i}$ > 200. This filtering strategy focuses the subsequent analyses on core genes, which are likely to function as hubs in the network and play critical roles in the regulatory mechanisms underlying ZGA in sheep. Following network filtering, the Louvain algorithm was employed for community detection. Initially, every gene was assigned to an individual community, and the quality of the resulting partition was evaluated based the modularity Q, defined for a weighted network as

$$Q=\frac{1}{2m}\sum_{i,j}\left[A_{ij}-\frac{k_{i}k_{j}}{2m}\right]\delta \left(c_{i},c_{j}\right),$$

where $c_{i}$ represents the community assignment of gene i and $\delta \left(c_{i},c_{j}\right)$ is the Kronecker delta function, which takes the value 1 when $c_{i}=c_{j}$ and 0 otherwise. For each node i, the algorithm calculates the change in modularity ΔQ associated with moving i from its current community to the community of one of its neighboring nodes. The modularity increment is calculated as

$$\Delta Q= [\frac{\sum \mathrm{in}+k_{i,in}}{2m}-{\left(\frac{\sum tot+k_{i}}{2m}\right)}^{2}]- [\frac{\sum in}{2m}-{\left(\frac{\sum tot}{2m}\right)}^{2}-{\left(\frac{k_{i}}{2m}\right)}^{2}]$$

A higher modularity Q indicates that interactions among genes within the same community are relatively dense, suggesting that these genes may participate in the same biological processes or regulatory networks [20]. Specifically, for each gene, the algorithm evaluates the change in modularity (ΔQ) that would result from moving the gene from its original community to one of its neighboring communities; only when ΔQ>0 is the gene reassigned, thereby gradually optimizing the network’s modular structure. After local optimization, the gene nodes within the same community are aggregated into “super-nodes” to construct a new aggregated network, and this process is iterated until the modularity converges, ultimately yielding a hierarchical community structure that reflects the regulatory characteristics of zygotic genome activation in sheep. The complete source code is available on GitHub (version 3.4.20) (https://github.com/miexiaochi/RNA_seq_data/tree/master, accessed on 5 June 2025).

### 2.7. Functional Annotation and Pathway Enrichment Analysis

Using R software (version 4.4.0), we performed GO enrichment analysis and KEGG pathway annotation for differentially expressed genes at various developmental stages using the clusterProfiler R package [21]. The GO enrichment analysis includes three categories: Cellular Component (CC), Molecular Function (MF), and Biological Process (BP).

## 3. Results

### 3.1. Summary of RNA-Seq Data

**Figure 1:**
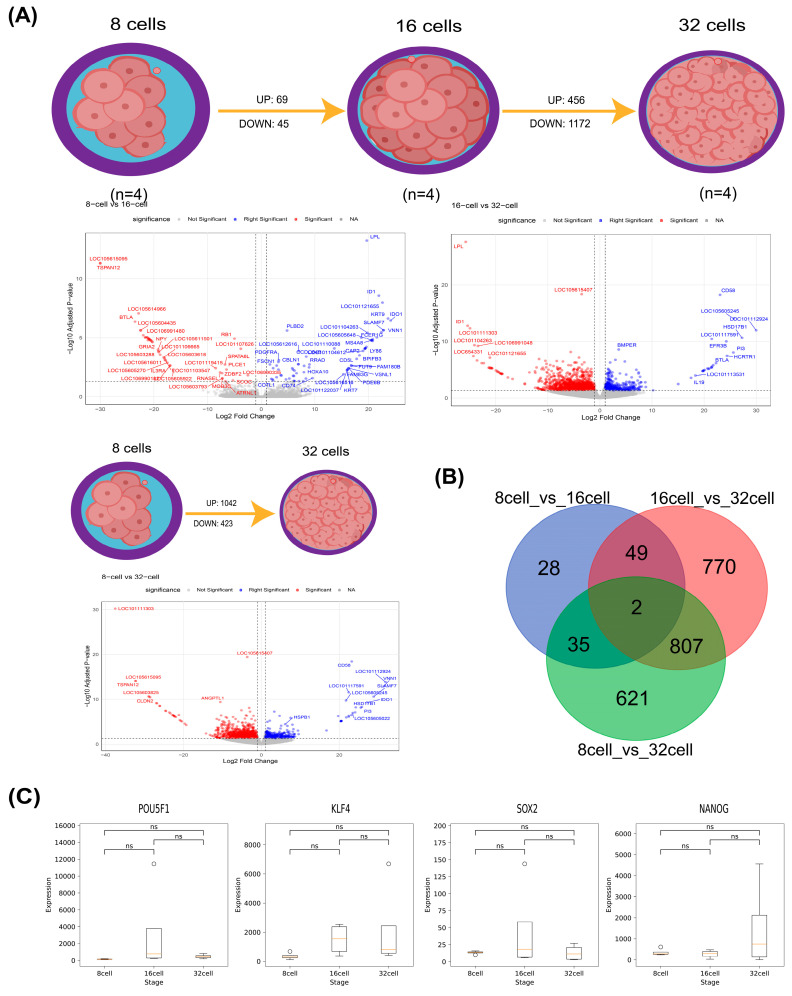
Analysis of significantly differentially expressed genes across different embryonic developmental stages. (**A**) Comparison of gene expression changes in the transitions 8- vs. 16-cell, 16- vs. 32-cell, and 8- vs. 32-cell, showing numbers of significantly upregulated (UP) and downregulated (DOWN) genes. (**B**) Venn diagram of stage-specific and shared differentially expressed genes among the 8-, 16-, and 32-cell stages. Numbers in each region indicate the counts of unique and overlapping DEGs. (**C**) Expression patterns of four core pluripotency transcription factors (SOX2, NANOG, POU5F1, KLF4) across the 8-, 16-, and 32-cell stages. Boxplots display the median and interquartile range (IQR); whiskers extend to 1.5× IQR, and hollow circles denote individual outlier values beyond this range. Pairwise *t*-test significance is annotated above each comparison (ns, not significant).

After quality trimming with Trimmomatic and alignment to the sheep reference genome using HISAT2, we obtained 299 GB of SAM-format reads. A summary of the RNA-Seq dataset, including the alignment metrics, is provided in Supplementary Table S1. Gene-level counts for 27,754 annotated genes were generated with FeatureCounts (Supplementary Table S2). Differential expression analysis with DESeq2 identified 114, 1628, and 1465 DEGs in the 8- vs. 16-cell, 16- vs. 32-cell, and 8- vs. 32-cell comparisons, respectively (Figure 1A; Supplementary Table S3). Strikingly, only two genes were shared across all three contrasts, indicating largely nonoverlapping transcriptional programs at each transition (Figure 1B). Notably, the core pluripotency factors *SOX2*, *NANOG, POU5F1,* and *KLF4* showed increased mean expression in the 16- vs. 32-cell transition, although paired t-tests did not reach significance—likely reflecting high intercellular variability at this stage (Figure 1C; Supplementary Table S4).

### 3.2. WGCNA Analysis Result

**Figure 2:**
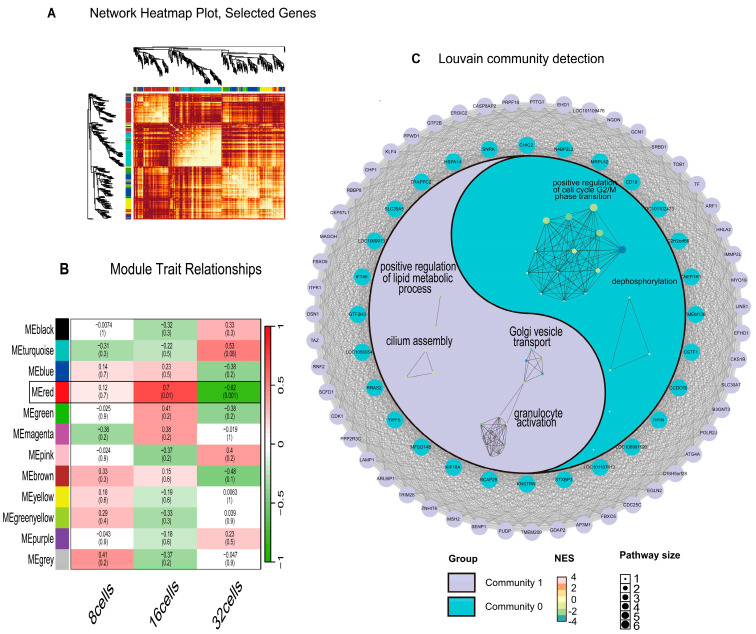
WGCNA and Louvain Community Detection Results. (**A**) Heatmap showing the predicted correlations among gene co-expression modules identified via WGCNA. (**B**) Module–trait relationship heatmap across embryonic developmental stages. The X-axis denotes the three developmental stages (8-cell, 16-cell, 32-cell), and the Y-axis lists the color-coded WGCNA gene modules. Each cell shows the Pearson correlation coefficient (upper number) between the module eigengene and stage, with the corresponding *p*-value in parentheses below. A continuous color bar alongside maps the correlation values from –1.0 (green) to +1.0 (red), facilitating a direct interpretation of the association strength and direction. (**C**) Circular plot illustrating the results of Louvain community detection and enrichment analysis. The outer ring displays genes from Community 1 (purple nodes), and the inner ring shows genes from Community 0 (light blue nodes). The central network represents functional enrichment results generated using the R package aPEAR, with node colors corresponding to the respective community.

We conducted WGCNA analysis on the counts matrix, encompassing gene clustering, module identification, and module interaction assessment (Figure 2A). This analysis identified a developmentally significant module, MEred, which is strongly associated with embryonic progression. The key genes within this module play a central regulatory role during the transition from the 16-cell to 32-cell stage (Figure 2B).

### 3.3. Louvain Community Detection Result

To dissect the internal modularity of the MEred-derived PPI network (Supplementary Table S5), we first filtered out low connectivity nodes (degree ≤ 200), yielding a core subnetwork of 182 genes linked by 1045 high-confidence edges. We then applied the Louvain algorithm for community detection, which partitions the network by optimizing modularity through iterative node reassignment and aggregation. This procedure identified two major communities, each corresponding to a distinct functional program during sheep ZGA (Figure 2C).

Community 0, composed of 89 genes, is functionally enriched in membrane trafficking and cytoskeletal dynamics. Central to this module is *TRAPPC2*, a core component of the *TRAPP* complex, which is essential for ER-to-Golgi vesicle tethering and fusion, facilitating early secretory pathway organization. Other prominent members, such as *BCAP29* and *YIPF5*, are involved in protein transport and vesicle docking, reinforcing this module’s role in intracellular trafficking. The presence of *KIF18A*, a kinesin motor protein that regulates microtubule-based chromosome alignment during mitosis, links membrane trafficking with cytoskeletal remodeling. Gene Ontology (GO) enrichment supports these functions, highlighting vesicle-mediated transport (GO:0006888, *p*.adj = 4.8 × 10^−5^), transmembrane transporter activity (GO:0022857, *p*.adj = 5.8 × 10^−2^), and microtubule motor activity (GO:0003777, *p*.adj = 2.6 × 10^−2^).

Community 1, encompassing 93 genes, converges on cell cycle regulation and RNA processing. This module is anchored by *CDK1* (Cyclin-dependent kinase 1), a master regulator of the G2/M transition, which phosphorylates key substrates to initiate mitotic entry. Other pivotal genes include *CDC25C*, which activates *CDK1* via dephosphorylation; *PTTG1*, a securin that prevents premature chromatid separation; and *FBXO5*, which inhibits the anaphase-promoting complex, ensuring proper cell cycle progression. These components drive robust enrichment in cell division (GO:0051301, *p*.adj = 9.6 × 10^−6^), mitotic cell cycle processes (GO:1903047, *p*.adj = 4.9 × 10^−4^), and oocyte meiosis (oas04114, *p*.adj = 1.6 × 10^−2^). Notably, RNA processing is tightly integrated into this regulatory axis through *PRPF18*, *MAGOH*, and *PPWD1*, which are core components of the spliceosome, mediating intron removal and mRNA maturation. GO terms such as spliceosomal complex (GO:0005681, *p*.adj = 2.1 × 10^−3^) and catalytic step 2 spliceosome (GO:0071013, *p*.adj = 3.5 × 10^−3^) confirm this role.

Together, these findings reveal a modular regulatory architecture underpinning sheep ZGA. Community 0 orchestrates membrane remodeling and cytoskeletal organization, facilitating the establishment of early secretory and signaling capacity, while Community 1 governs cell cycle entry and transcriptome maturation, thereby supporting precise coordination of nuclear and cytoplasmic events during the maternal-to-zygotic transition

### 3.4. Results of Functional Annotation and Pathway Enrichment Analysis

**Figure 3:**
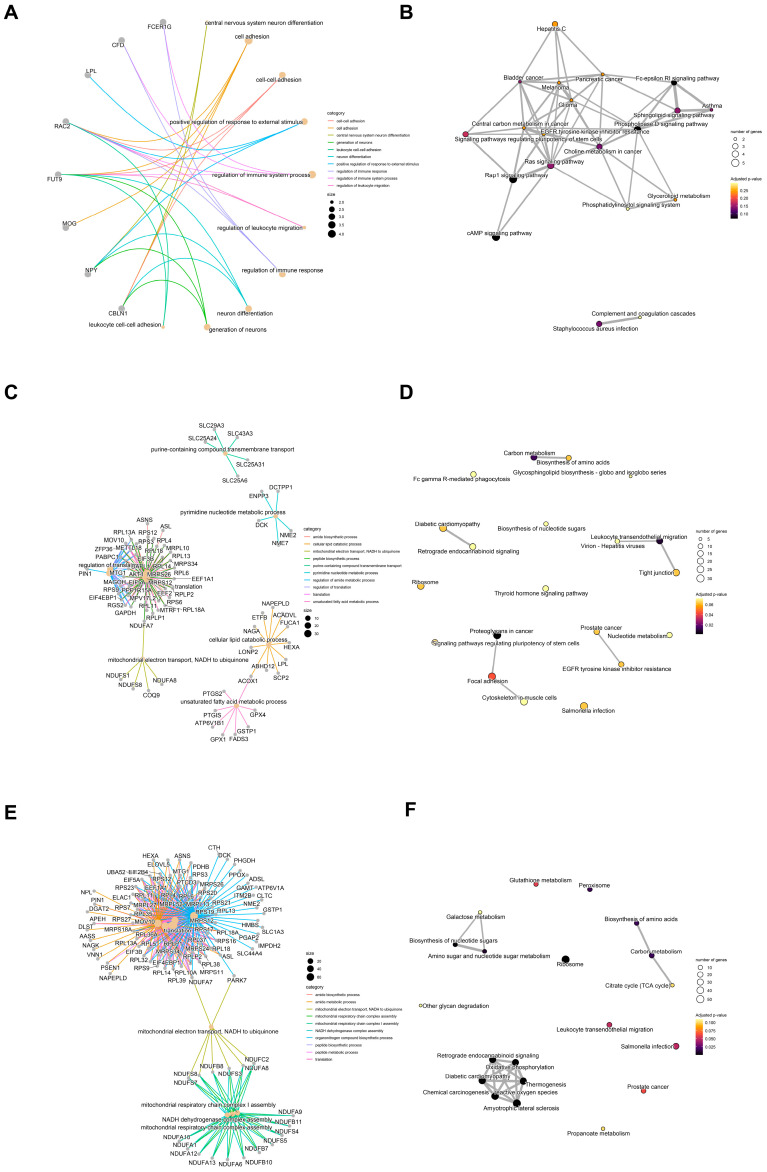
Functional enrichment analysis of differentially expressed genes during early embryonic development stages. (**A**) GO enrichment analysis of differentially expressed genes between the 8-cell stage and the 16-cell stage. (**B**) KEGG enrichment analysis of differentially expressed genes between the 8-cell stage and the 16-cell stage. (**C**) GO enrichment analysis of differentially expressed genes between the 16-cell stage and the 32-cell stage. (**D**) KEGG enrichment analysis of differentially expressed genes between the 16-cell stage and the 32-cell stage. (**E**) GO enrichment analysis of differentially expressed genes between the 8-cell stage and the 32-cell stage. (**F**) KEGG enrichment analysis of differentially expressed genes between the 8-cell stage and the 32-cell stage.

In the comparison of 8 vs. 16 cells (Figure 3A,B), GO enrichment revealed that processes such as “neuronal differentiation of the central nervous system” (GO:0021953), “cell adhesion” (GO:0007155), and “cell-cell adhesion” (GO:0098609) were significantly enriched. The corresponding differentially expressed genes included CBLN1, NPY, MOG, FUT9, RAC2, etc. This result indicates that at the 16-cell stage, genes related to neuronal differentiation in the embryonic transcriptome begin to be expressed, and intercellular adhesion molecules gradually establish, laying the foundation for the subsequent formation of cell polarity and tissue morphology (Figure 3A). The KEGG enrichment analysis showed that the “Rap1 signaling pathway” (bta04015) and the “cAMP signaling pathway” (bta04024) were marginally significantly different (*p*.adjust ≈ 0.068) at the 8–16 stage. Rap1 and cAMP signaling play a crucial role in embryonic cell proliferation, migration, and differentiation. Their early activation indicates that the cell signaling network begins to mediate phenotypic shaping after ZGA (Figure 3B). In the comparison of 16 vs. 32 cells (Figure 3C,D), the GO enrichment of “translation” (GO:0006412), “regulation of translation” (GO:0006417), and “peptide biosynthesis process” (GO:0043043) was significantly enriched. The corresponding differentially expressed genes included EIF4EBP1, EIF3B, MRPS34, RPLP2, AKT1, etc. In the KEGG pathway, “ribosome” (bta03010) showed extremely significant enrichment (*p*.adjust ≈ 2.37 × 10^−18^), and 52 out of 551 differentially expressed genes were members of the ribosomal protein family. This result indicates that the protein translation capacity of the 16- vs. 32-cell phase has reached its peak, providing a foundation for rapid cell proliferation and functional differentiation, which is consistent with the extensive mRNA translation and ribosome assembly during the main ZGA period (Figure 3C). The KEGG pathway “oxidative phosphorylation” (bta00190, 33/551 genes, *p*.adjust ≈ 1.37 × 10^−8^) and “carbon metabolism” (bta01200, 19/611 genes, *p*.adjust ≈ 0.0135) are significantly enriched, clearly indicating a significant enhancement in the mitochondrial energy supply. During this period, the cell proliferation rate accelerated, requiring a higher ATP output and the need for energy metabolism reprogramming in the early stage of development to adapt to rapid proliferation (Figure 3D). For the 8- vs. 32-cell overall comparison, protein synthesis and mitochondrial function continue to strengthen; genes related to neuronal differentiation and cell adhesion are gradually expressed; the signal transduction network and metabolic pathways jointly regulate, demonstrating the dynamic coupling of transcription, translation, and metabolism during embryonic development (Figure 3E,F).

In the 16-cell to 32-cell comparison, a substantial increase in transcriptomic activity was observed (n = 1628 DEGs). However, multiple enriched GO terms lost statistical significance after multiple testing correction, likely due to functional heterogeneity (Figure 3C). Nonetheless, the enrichment of terms such as granulocyte activation (GO:0036230), leukocyte activation involved in immune response (GO:0002366), and regulation of cell morphogenesis (GO:0022604) was detected (Figure 3D). KEGG analysis identified enrichment in pathways such as proteoglycans in cancer (oas05205), tight junction (oas04530), and endocytosis (oas04144), indicating the activation of cell adhesion, cytoskeletal remodeling, and vesicular trafficking programs critical for morphogenesis and compaction during this transition (Figure 3C,D).

The 8-cell to 32-cell comparison, which reflects the cumulative transcriptional changes across early ZGA, yielded a broad spectrum of enriched terms predominantly related to biosynthetic and metabolic processes. Notably, GO terms such as amide metabolic process (GO:0043603), translation (GO:0006412), cellular respiration (GO:0045333), and oxidative phosphorylation (GO:0006119) were significantly overrepresented (adjusted *p* < 1 × 10^−7^) (Figure 3E,F). KEGG pathway analysis consistently demonstrated enrichment in metabolism-centered pathways, including carbon metabolism (oas01200), biosynthesis of nucleotide sugars (oas01250), inositol phosphate metabolism (oas00562), and oxidative phosphorylation (oas00190), suggesting a developmental shift toward enhanced translational output and mitochondrial bioenergetics to support post-ZGA proliferation and morphogenesis (Figure 3E,F).

Collectively, these results indicate that early-stage DEGs (8 vs. 16) are functionally linked to signaling and immune priming, whereas later-stage DEGs (16 vs. 32 and 8 vs. 32) are primarily engaged in protein biosynthesis, energy metabolism, and organelle maturation. This stage-specific transition in molecular function reflects the progressive reprogramming of embryonic cells during ZGA and provides functional insight into the transcriptional logic of early sheep development.

## 4. Discussion

### 4.1. Two-Stage Regulatory Mechanisms of Sheep ZGA

Sheep zygotic genome activation (ZGA) proceeds not as an abrupt switch but as a two-phase continuum. During the minor ZGA phase (8-cell vs. 16-cell transition), only 114 genes are differentially expressed (Figure 1A), yet these include key signaling and patterning factors such as *NODAL*, *HOXA10*, and *ID1*. Gene Ontology enrichment highlights innate immune readiness and intercellular signaling (“response to other organism,” “defense response”; Figure 3B), suggesting that early embryos prime essential communication and developmental pathways before widespread transcription begins. Notably, similar early immune- and signaling-related gene activations have been observed during minor ZGA in bovine embryos, whereas murine minor ZGA primarily primes cell cycle and RNA-processing genes [1,2]. Thus, while the two-phase logic appears to be conserved, sheep early embryos emphasize immune-related functions more strongly, perhaps reflecting species-specific requirements for controlling the maternal environment. This selective transcription may establish the framework for subsequent pluripotency network activation.

In the major ZGA phase (16- vs. 32-cell transition), transcriptomic activity expands dramatically: 1628 DEGs are detected (Figure 1A), including the core pluripotency transcription factors *SOX2, NANOG, POU5F1*, and *KLF4* (Figure 1B). Concomitantly, the DNA demethylase *TET1* is upregulated, indicating an active epigenetic reprogramming that erases maternal methylation marks to enable robust embryonic transcription (Supplementary Figure S1). The surge in the DEG count and the enrichment of cell-cycle and RNA-processing pathways underscore the global remodeling of gene regulatory networks as the embryo assumes transcriptional autonomy [22,23].

Notably, a total of 1 742 transcription factors exhibit dynamic, stage-specific expression changes when both transitions are considered, reflecting finely tuned regulatory waves that coordinate minor and major ZGA. Collectively, these data support a two-stage model in which an initial priming phase selectively engages signaling and patterning genes, followed by a broad activation phase driven by core pluripotency factors and epigenetic modifiers. Future studies should dissect the upstream regulators that trigger each phase and employ targeted functional assays (e.g., qRT-PCR, loss-of-function) to validate candidate drivers of sheep ZGA.

### 4.2. Core Pluripotency Transcription Factors in Sheep Zygotic Genome Activation

*SOX2*, *NANOG*, *POU5F1*, and *KLF4*, as essential core pluripotency transcription factors, play pivotal roles in embryonic development across various species [24,25]. The core pluripotency transcription factors (TFs) *NANOG*, *SOX2*, and *OCT4* collaboratively regulate the intrinsic pluripotency transcription factor network [26]. Moreover, pluripotent embryonic stem cells express “auxiliary” pluripotency regulators, such as *KLF4*, a process also reflected by our findings (Figure 1B). In sheep embryonic development, our research reveals a marked upregulation of *POU5F1* during ZGA, suggesting its involvement in the transition of sheep embryos from maternal dependence to zygotic genome activation. *SOX2* also plays a significant role in embryonic development. In sheep embryos, changes in its expression may be closely related to the formation and development of specific cell types. *NANOG* is vital for maintaining the self-renewal and pluripotency of embryonic stem cells. Its upregulation during sheep zygotic genome activation may provide the necessary conditions for the subsequent differentiation of totipotent embryonic stem cells [27,28,29,30]. In this study, the significant changes in core pluripotency transcription factors during sheep ZGA further confirm their central roles in regulating the ZGA process.

### 4.3. Modular Logic of PPI–Louvain Integration

In the present study, we extended the traditional co-expression analysis by overlaying the MEred-derived gene set onto a high-confidence protein–protein interaction (PPI) network, thereby integrating transcriptional correlation with physical association data. While WGCNA effectively clusters genes according to shared expression profiles, it cannot discriminate between direct biochemical interactions and indirect, co-regulated relationships; by incorporating PPI information from the STRING v11 database, we leverage experimentally validated and computationally predicted associations to resolve mechanistic cores within each module [31]. This integrative strategy is grounded in the principles of network biology, which posit that cellular functions emerge from the coordinated action of interacting proteins and that PPI networks can reveal functional assemblies not apparent from expression data alone [32].

To partition this combined co-expression/PPI network into biologically coherent substructures, we applied the Louvain algorithm, a two-phase, greedy optimization method designed to maximize network modularity [33]. In the first phase, individual nodes are iteratively reassigned to neighboring communities if doing so yields a positive gain in modularity, effectively capturing local edge density enhancements. In the second phase, entire communities are collapsed into “super-nodes,” and the process repeats, uncovering a hierarchical organization of communities at multiple scales. This approach has been shown to efficiently and accurately identify community structures in large and complex networks, outperforming earlier methods in both speed and modularity quality [34].

Recognizing that peripheral or spurious interactions can obscure core regulatory modules, we filtered the PPI network to retain only nodes with a degree > 200 before community detection. This thresholding enriches for hub-centered subnetworks, which, in accordance with network biology theory, often correspond to essential regulatory circuits and molecular complexes [32]. Indeed, a hub-based analysis has been demonstrated to enhance the detection of functional modules in both health and disease contexts, ensuring that resulting communities represent robust, high-confidence interaction cores rather than noise-driven clusters [35,36,37,38].

Biologically, the two communities delineated by Louvain detection map onto the major functional demands of early embryogenesis. The first community, enriched for vesicle-mediated transport and cytoskeletal dynamics, likely orchestrates the establishment of the secretory pathways and membrane architecture necessary for signal reception and organelle biogenesis during ZGA. The second community, characterized by cell-cycle regulators and spliceosomal components, appears to coordinate the G2/M transition and mRNA processing modules essential for accurate cell division and transcriptome maturation. This clear division of labor underscores how discrete network modules can simultaneously satisfy the sequential and overlapping requirements of zygotic genome activation [6,39,40,41].

Methodologically, our multi-layered framework—combining WGCNA and PPI integration with Louvain community detection—mitigates the limitations inherent to each individual approach. Spurious co-expression links are filtered by physical interaction evidence, while community detection distills complex networks into functionally coherent modules. Such integrative network methodologies are increasingly recognized as critical for unraveling the architecture of developmental processes, from molecular assemblies to higher-order morphogenetic events [42,43,44]. Future extensions could incorporate temporally resolved PPIs or single-cell network reconstructions to capture the dynamic reconfiguration of regulatory modules as embryos progress through ZGA.

## 5. Conclusions

This study provides a systems-level view of sheep zygotic genome activation by integrating differential expression, WGCNA, and PPI-guided community detection.

We identified stage-specific transcriptional changes, highlighted core pluripotency factors, and uncovered two major regulatory communities coordinating membrane dynamics and cell cycle progression.

These findings offer new insights into the modular regulatory architecture underlying early embryogenesis in sheep.

## Data Availability

The datasets generated and/or analyzed during the current study are not publicly available due to privacy and proprietary constraints but are available from the corresponding author on reasonable request.

## Supplementary Materials

The following supporting information can be downloaded at: https://www.mdpi.com/article/10.3390/biology14060676/s1.
